# Chronic alcohol intake disrupts cytochrome P450 enzyme activity in alcoholic fatty liver disease: insights into metabolic alterations and therapeutic targets

**DOI:** 10.3389/fchem.2025.1509785

**Published:** 2025-05-13

**Authors:** Qian Zhu, Xuefeng Xie, Ling Fang, Cheng Huang, Jun Li

**Affiliations:** ^1^ Inflammation and Immune Mediated Diseases Laboratory of Anhui Province, Anhui Institute of Innovative Drugs, School of Pharmacy, Anhui Medical University, Hefei, China; ^2^ The First Affiliated Hospital of Anhui Medical University, Hefei, China

**Keywords:** alcoholic fatty liver, chronic ethanol intake, cytochrome P450, rat model, liver alcohol

## Abstract

**Introduction:**

Alcoholic fatty liver disease (AFLD) is a common consequence of chronic alcohol consumption, characterized by lipid accumulation and oxidative stress in the liver. Cytochrome P450 (CYP450) enzymes play essential roles in metabolizing alcohol and other compounds. However, the specific long-term effects of alcohol on these enzymes remain unclear.

**Methods:**

This study the examines influence of prolonged ethanol exposure on CYP450 activity and expression in AFLD using a rat model. Key enzymes such as CYP2E1, CYP2D6, and CYP3A1 were assessed in relation to lipid accumulation and oxidative stress.

**Results:**

Significant alterations were identified in the expression and activity of CYP2E1, CYP2D6, and CYP3A1, which were associated with increased lipid accumulation and oxidative stress in the liver. Additionally, the expression of P-glycoprotein (P-gp) was elevated, suggesting that chronic alcohol intake may impact drug transport and excretion.

**Discussion:**

These findings provide new insights into the molecular mechanisms of AFLD and highlight the potential of CYP450 modulation as a therapeutic target. By elucidating how long-term ethanol exposure disrupts hepatic CYP450 enzyme profiles, this research lays the groundwork for developing personalized therapeutic strategies to improve outcomes for patients with AFLD.

## 1 Introduction

Alcoholic Fatty Liver Disease (AFLD) is a disease closely associated with chronic alcohol consumption that can progress from simple fatty liver to cirrhosis and even liver cancer (Seitz et al., 2018; Fang et al., 2022; Kong et al., 2021; Zhang et al., 2023a). This progression involves intricate biochemical and molecular mechanisms, with the cytochrome P450 (CYP450) system playing a crucial role in alcohol metabolism (Coon and Koop, 1987; Rabelo et al., 2024; Yang et al., 2021). The CYP450 enzyme system comprises a diverse group of enzymes present in the liver and other tissues, involved not only in alcohol metabolism but also in the metabolism of various drugs and exogenous compounds (Zhao et al., 2021; Lu and George, 2024; Harjumäki et al., 2021; Li et al., 2023a). However, the precise role of CYP450 in AFLD remains unclear (Rabelo et al., 2024). Recent studies have begun to unveil the significance of changes in CYP450 enzyme activity and expression during the development of AFLD (Krishnarjuna et al., 2024). For instance, research indicates a significant increase in the expression of Cytochrome P450 2E1 (CYP2E1) in an AFLD rat model following prolonged alcohol exposure, linked to increased lipid accumulation and oxidative stress (LI et al., 2022; Li et al., 2024; Nasrin et al., 2021; Kang et al., 2022). Additionally, alterations in CYP3A4 have been associated with abnormal drug metabolism in AFLD patients, suggesting that modulating the CYP450 enzyme system may be a key factor in the pathogenesis of AFLD (Srivatsa, 2022). Deeper investigations reveal that different subtypes of CYP450 enzymes play unique roles at various stages of AFLD development, with changes in some subtypes’ activity serving as potential biomarkers for early AFLD detection (Luo et al., 2023; Iversen et al., 2022; Gougis et al., 2021; Bansal et al., 2023). For example, overexpression of CYP4A11 has been closely linked to inflammation and fibrosis progression in AFLD patients, indicating a possible regulatory role in disease advancement (Dai et al., 2023; Murase et al., 2023; Wenzel et al., 2023). These findings underscore the multifaceted role of CYP450 enzymes in AFLD, extending beyond their involvement in alcohol metabolism to regulate lipid metabolism, oxidative stress, and inflammatory responses during disease progression (Yang et al., 2021; Harjumäki et al., 2021). Therefore, comprehending the role of CYP450 enzymes in AFLD is crucial for unraveling the disease’s molecular mechanisms and developing new diagnostic and therapeutic strategies (Jiang et al., 2024; Rabelo et al., 2024).

Long-term alcohol exposure can significantly change the expression and activity of CYP450 enzymes in liver cells (Coon and Koop, 1987). The metabolism of alcohol in the liver involves multiple subtypes of CYP450, including CYP2E1, CYP1A2, and CYP3A1 (Zakhari, 2006; Xu et al., 2022; Wang et al., 2021a). CYP2E1 has garnered widespread attention due to its crucial role in ethanol oxidation (Katary and Abdel-Rahman, 2020). The increased activity of CYP2E1 not only accelerates alcohol metabolism but also leads to a significant production of reactive oxygen species (ROS) (Chen et al., 2021; Hussain et al., 2022; Harjumäki et al., 2021). By damaging the lipid membrane, proteins, and DNA, these ROS trigger intracellular oxidative stress responses and promote inflammatory reactions and cell death, exacerbating liver cell damage (Kong et al., 2024; Jiang et al., 2022). Moreover, oxidative stress can activate hepatic stellate cells, promoting the development of liver fibrosis, which is a crucial step in the occurrence of cirrhosis and liver cancer. CYP1A2 plays a role in processing alcohol metabolites such as polycyclic aromatic hydrocarbons, and changes in its activity may affect the liver’s detoxification capacity for these harmful substances, thereby impacting liver health (Eberhardt et al., 2023). Meanwhile, CYP3A1 (corresponding to CYP3A4 in humans), as one of the most abundant subtypes of CYP450 in the liver, is involved in the metabolism of multiple drugs (Li et al., 2023b; Muhamad et al., 2022). Alcohol-induced changes in CYP3A1 activity not only influence the clearance rate of drugs but may also alter the efficacy and toxic side effects of drugs, especially those primarily metabolized by CYP3A4 (Iwaki et al., 2018; Wang et al., 2019). This impact may necessitate adjustments to drug treatment regimens to avoid adverse reactions or drug interactions (Singh et al., 2023). Changes in the activity of these enzymes may affect the oxidation and detoxification processes of alcohol, leading to the generation of free radicals, excessive lipid accumulation, oxidative stress, and further liver damage (Massart et al., 2022). Furthermore, alterations in CYP450 enzymes may also impact the metabolism of other drugs and exogenous compounds, increasing patients’ risks of adverse reactions (Zhao et al., 2021; Zhai et al., 2023; Liu et al., 2021).

The rat model is a common experimental animal model used to study human diseases, particularly suitable for AFLD research (Zhang et al., 2022; Wang et al., 2021b). Establishing a rat model with alcohol feeding can mimic liver changes induced by long-term alcohol exposure in humans; while the existing rat models of AFLD can mimic liver changes induced by long-term alcohol exposure in humans, they have certain limitations in accurately replicating the complex liver alterations caused by prolonged alcohol consumption (Lau et al., 2024; Yan et al., 2023a; Lu et al., 2023). These limitations mainly stem from the singular alcohol administration pattern and the inadequate simulation of the complexity of human drinking behavior, which may affect the model’s effectiveness and the generalizability of research outcomes (Pascale et al., 2022; Vujkovic et al., 2022; Luo et al., 2021). To address these shortcomings in existing models, this study adopts innovative methods to more accurately simulate the impact of human alcohol consumption on the liver. Firstly, by implementing a multi-stage alcohol administration strategy that gradually increases alcohol concentration, mimicking the natural progression of alcohol intake over time in humans, the study aims to closely resemble real alcohol exposure processes. Additionally, by incorporating specific nutritional interventions to account for the influence of dietary factors on AFLD development, the model’s authenticity and applicability are further enhanced. The study further observes the dynamic changes in CYP450 enzyme activity and expression. However, research on how long-term alcohol exposure affects CYP450 enzyme activity in AFLD rat models remains relatively limited, with differing viewpoints and conclusions.

This research aims to delve into the specific changes in liver CYP450 enzyme activity and expression during the progression of AFLD by establishing a rat model that mimics prolonged human alcohol intake conditions. Compared to previous studies, comprehensive experimental designs are employed to more precisely elucidate the regulatory mechanisms of the CYP450 enzyme system in AFLD development. It is particularly crucial since although the critical role of the CYP450 enzyme system in alcohol metabolism and liver diseases is known, its specific role in the AFLD process remains incompletely understood. Our study fills knowledge gaps in this area and offers a new perspective on how alcohol influences liver function and drug metabolism by regulating these key enzymes, providing the scientific groundwork for developing novel therapeutic strategies for AFLD, particularly in identifying and validating new drug targets. By gaining a deeper understanding of the role of CYP450 enzymes in AFLD, we aim to advance the development and optimization of AFLD treatment strategies, offering more effective interventions and treatment options for patients with this serious liver disease.

## 2 Results

### 2.1 Identification of key differential genes influencing AFLD in rats through bioinformatics analysis

**FIGURE 1:**
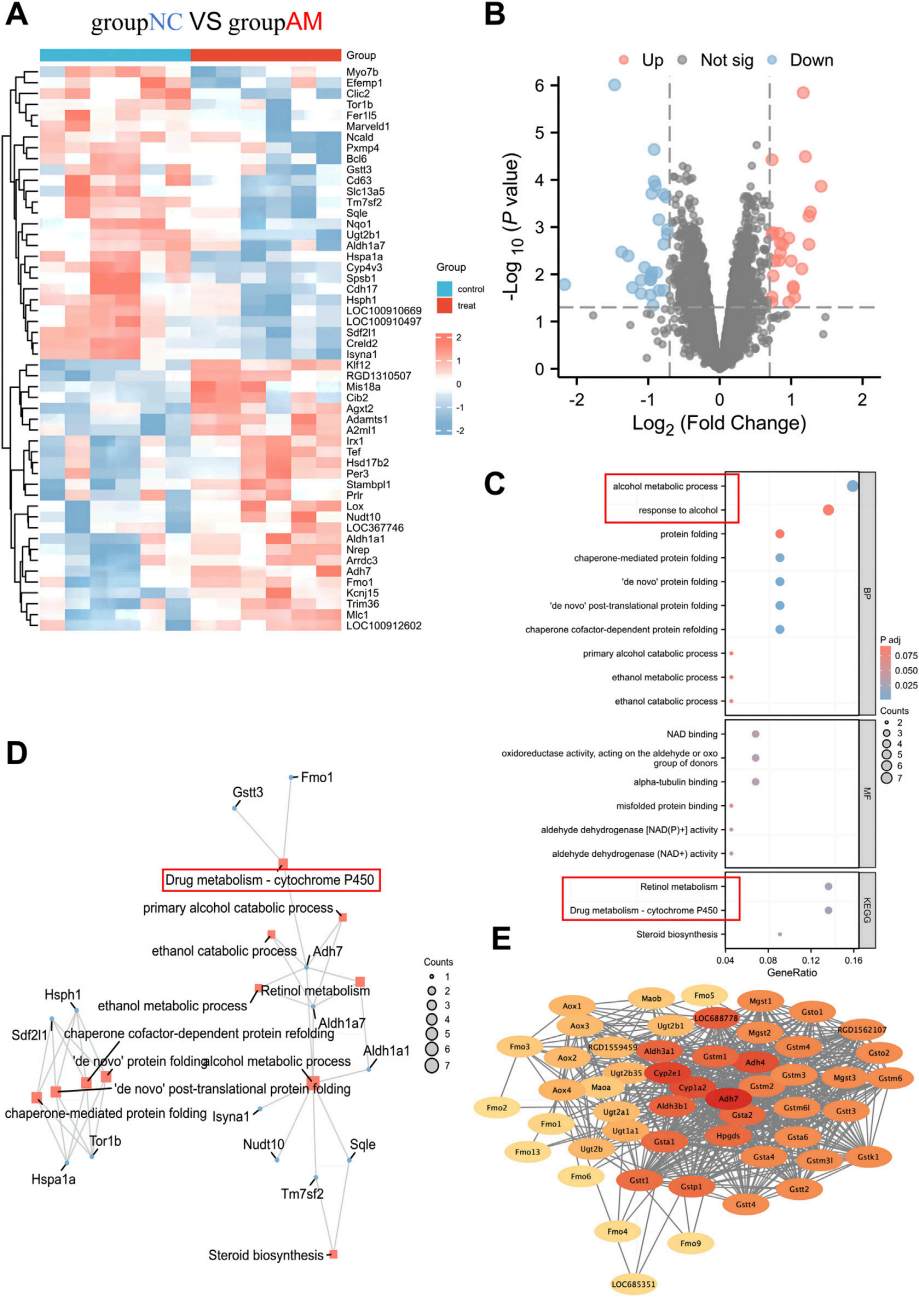
Bioinformatics analysis identifying key differential genes affecting rat AFLD. Note: **(A)** Heatmap of differential gene expression between normal and fatty liver groups, where each row represents a gene and each column a sample. The intensity of the color indicates the level of gene expression, with red denoting high expression and blue low expression. **(B)** Volcano plot of differential analysis between normal and fatty liver groups. The *x*-axis represents Log2 (Fold Change), and the *y*-axis represents −Log(P value). Red dots indicate genes with high expression, and blue dots indicate low expression. **(C)** Bubble chart of GO and KEGG enrichment analysis results, where the *x*-axis shows the GeneRatio and the *y*-axis the enriched entries. The size of the bubble indicates the number of genes involved in the pathway, and the color of the bubble indicates the p-value. Enriched pathways involve biological processes, including chaperone cofactor-dependent protein refolding, alcohol metabolic process, ethanol catabolic process, and response to alcohol, and pathways such as Drug metabolism - CYP450, Steroid biosynthesis, and Retinol metabolism. **(D)** The gene-pathway network diagram shows the relationships between DEGs and enriched pathways, with nodes representing genes or pathways and edges indicating their associations. **(E)** Network diagram of the Drug metabolism - CYP450 gene interactions, where the intensity of the color indicates the Degree, with darker colors representing higher degrees.

Differential analysis was conducted on the gene expression profiles of 6 healthy control and 6 AFLD rats from the GSE66316 dataset, revealing 52 DEGs (Figure 1A). The complete list of DEGs is provided in Supplementary Table S1, and a heatmap of the 52 DEGs is shown in Figure 1B. GO and KEGG enrichment analyses of the DEGs revealed their involvement in biological processes such as chaperone cofactor-dependent protein refolding, alcohol metabolic process, ethanol catabolic process, and response to alcohol. The enriched pathways mainly included drug metabolism - CYP450, Steroid biosynthesis, and retinol metabolism (Figure 1C). Based on the enrichment analysis findings, a gene-pathway network diagram was generated (Figure 1D), highlighting the Drug metabolism - CYP450 pathway of particular interest due to its relevance to alcohol metabolism. Further exploration through the STRING database identified key genes Adh7, CYP2E1, Adh4, and Cyp1a2 at the network’s core, as indicated by their connectivity within the network diagram (Figure 1E). These results suggest that genes such as ADH7, CYP2E1, ADH4, and CYP1A2 may influence AFLD in rats through the Drug metabolism - CYP450 pathway.

### 2.2 Successful establishment of AFLD rat model

**FIGURE 2:**
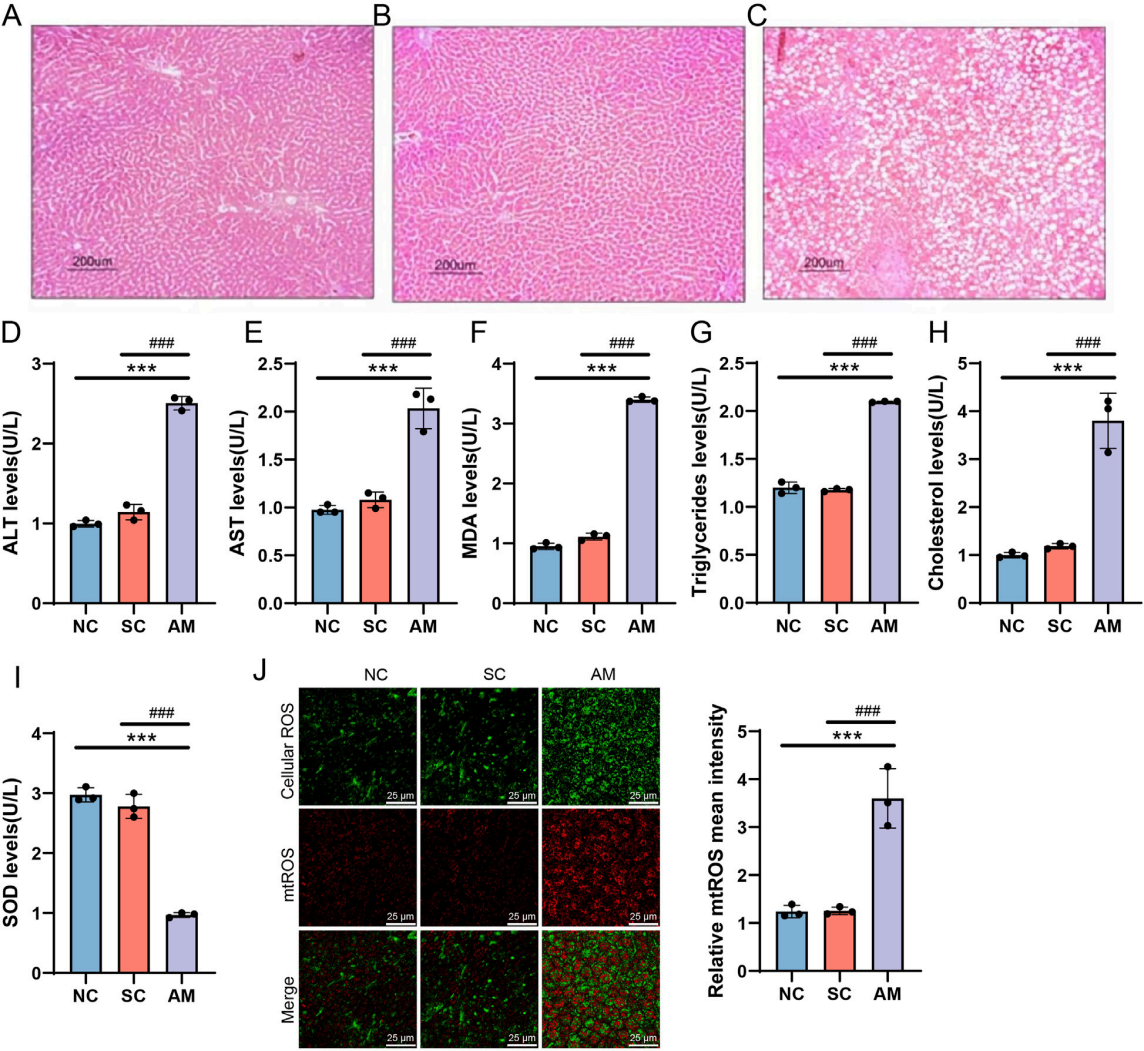
Histological changes in the livers of AFLD rats (H&E staining, scale bar = 200 μm). Note: **(A)** Normal control group, showing no significant fat accumulation. **(B)** The sucrose solution group showed no fat droplets. **(C)** The ethanol intake group showed severe tissue vacuolation and significant fat droplet accumulation. **(D–I)** Serum biochemical analysis; **(J)** ROS detection. Compared to the normal control group, * indicates P < 0.05, ** indicates P < 0.01, and *** indicates P < 0.001, indicating significant differences as determined by the least significant difference (LSD) test. Compared to the sucrose solution group, # indicates P < 0.05, ## indicates P < 0.01, and ### indicates P < 0.001, also determined by the LSD test. All experiments were repeated three times.

In the livers of rats subjected to chronic ethanol intake, macrovesicular steatosis and significant hepatic lipid accumulation were observed. These changes were visually evident through histological analysis using H&E staining. The NC group exhibited lobular structure without noticeable fat deposition (Figure 2A). No lipid droplets were observed in the SC group (Figure 2B). In contrast, the AM group displayed macrovesicular steatosis, leading to severe hepatic lipid accumulation (Figure 2C). The detection of serum biochemical indicators showed that ALT and AST, MDA levels, triglyceride (TG) and total cholesterol (TC) levels were significantly increased in AFLD rats, while SOD levels were significantly decreased (Figures 2D–I). Furthermore, ROS level measurements indicated that oxidative stress was exacerbated in AM group rats, with a significant increase in intracellular ROS levels (Figure 2J). These histopathological features are consistent with the typical presentation of AFLD (Gao et al., 2022), validating the successful establishment of the rat model.

### 2.3 Changes in AFLD rat liver P450 mRNA and protein expression levels

**FIGURE 3:**
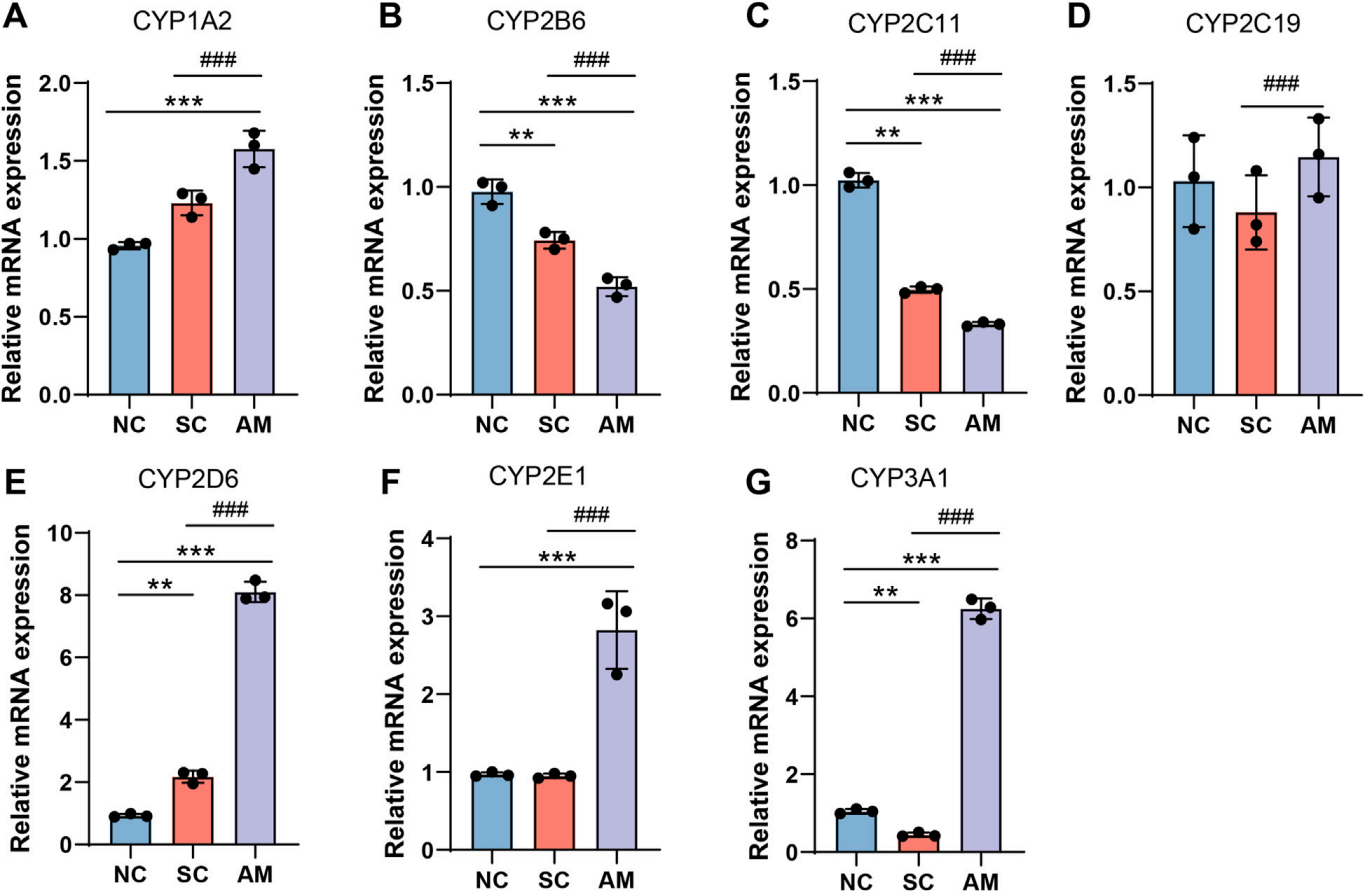
Relative mRNA expression levels of CYP450 enzymes in the livers of AFLD rats. Note: **(A)** mRNA expression of CYP1A2 in rat liver tissue; **(B)** mRNA expression of CYP2B6 in rat liver tissue; **(C)** mRNA expression of CYP2C11 in rat liver tissue; **(D)** mRNA expression of CYP2C19 in rat liver tissue; **(E)** mRNA expression of CYP2E1 in rat liver tissue; **(F)** mRNA expression of CYP3A1 in rat liver tissue; **(G)** mRNA expression of CYP3A1 in rat liver tissue. Compared to the normal control group, * indicates P < 0.05, ** indicates P < 0.01, and *** indicates P < 0.001, indicating significant differences as determined by the least significant difference (LSD) test. Compared to the sucrose solution group, # indicates P < 0.05, ## indicates P < 0.01, and ### indicates P < 0.001, also determined by the LSD test. All experiments were repeated three times.

As depicted in Figure 3, the mRNA levels of CYP1A2, CYP2D6, CYP2E1, and CYP3A1 in the rat liver were significantly higher in the AM group compared to the NC and SC groups. Conversely, the mRNA levels of CYP2B6 and CYP2C11 were significantly lower in the AM group when compared to the NC and SC groups. The mRNA expression of CYP2C19 showed a significant difference across all groups.

**FIGURE 4:**
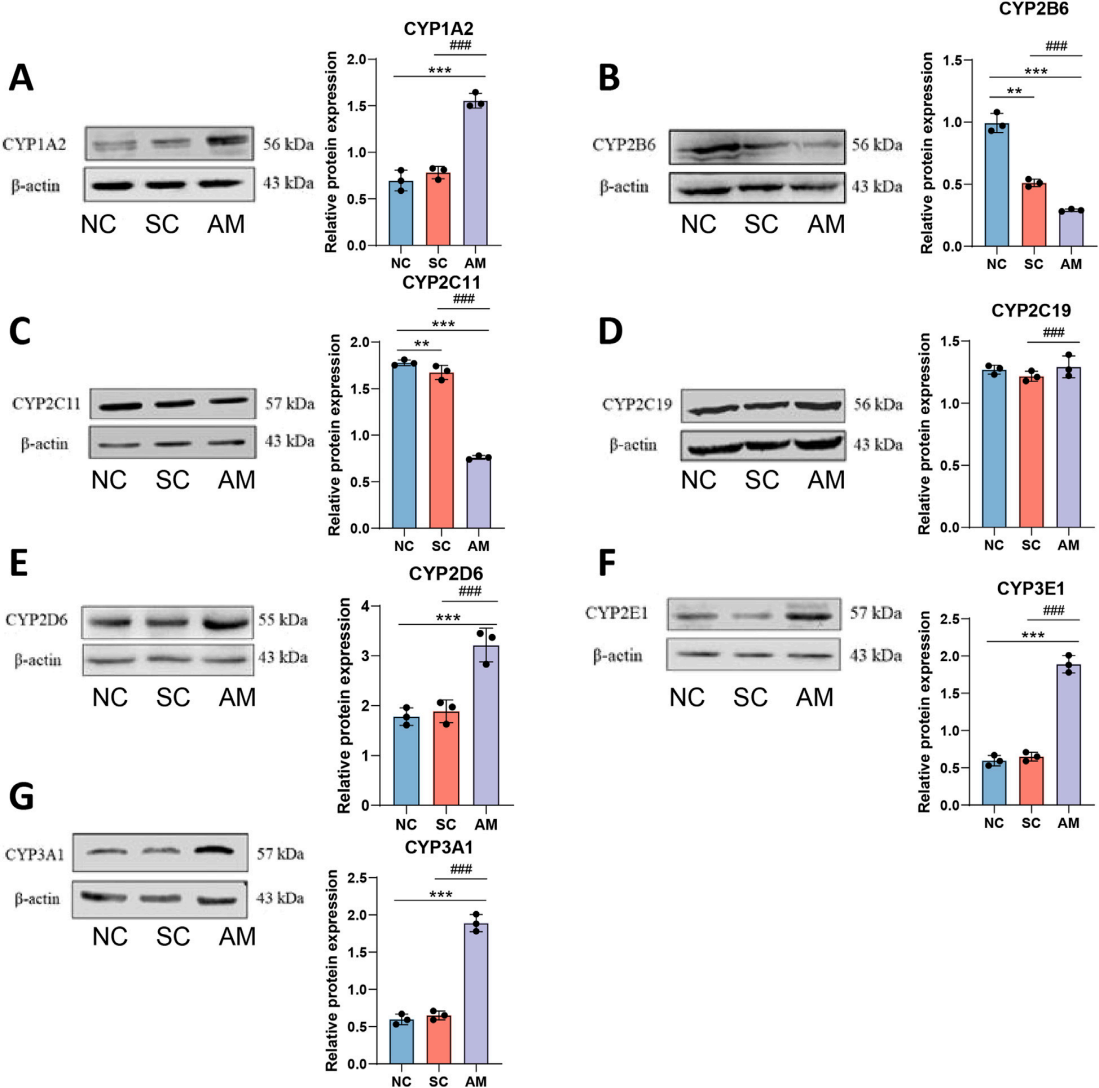
Relative protein expression levels of CYP450 enzymes in the livers of AFLD rats. Note: **(A)** Protein level of CYP1A2 in rat liver tissue; **(B)** Protein level of CYP2B6 in rat liver tissue; **(C)** Protein level of CYP2C11 in rat liver tissue; **(D)** Protein level of CYP2C19 in rat liver tissue; **(E)** Protein level of CYP2D6 in rat liver tissue; **(F)** Protein level of CYP3E1 in rat liver tissue; **(G)** Protein level of CYP3A1 in rat liver tissue. Compared to the normal control group, * indicates P < 0.05, ** indicates P < 0.01, and *** indicates P < 0.001, indicating significant differences as determined by the LSD test. Compared to the sucrose solution group, # indicates P < 0.05, ## indicates P < 0.01, and ### indicates P < 0.001, also determined by the LSD test. All experiments were repeated three times.

Furthermore, using techniques such as Western Blot, we quantified the expression levels of these enzymes. As shown in Figure 4, consistent with the mRNA results, the protein expression of CYP1A2, CYP2D6, CYP2E1, and CYP3A1 was higher in the AM group compared to the NC and SC groups. Conversely, the protein levels of CYP2B6 and CYP2C11 were significantly lower in the AM group compared to the NC and SC groups. There was no significant difference in the protein expression of CYP2C19 across all groups. The elevation in mRNA and protein expression levels of CYP1A2, CYP2D6, CYP2E1, and CYP3A1 reflects increased liver metabolic activity due to alcohol exposure, particularly in detoxification and alcohol metabolism processes. These changes may represent an adaptive response of the liver to continuous alcohol intake by enhancing the expression of specific CYP450 enzymes to accelerate the clearance of alcohol and its toxic metabolites. This enhanced metabolic adaptive response may have a protective role in the early stages of AFLD, but in the long term, it may lead to increased oxidative stress and liver damage, especially as CYP2E1 is known to generate high levels of ROS, exacerbating oxidative stress.

On the contrary, the decreased protein expression of CYP2B6 and CYP2C11 may indicate a specific negative impact of alcohol intake on liver metabolic capability, resulting in impaired metabolic functions of these enzymes. CYP2B6 and CYP2C11 play roles in the metabolism of various drugs and foreign compounds, and the decrease in their expression levels may slow down the metabolism of these substances, increasing the risk of liver toxicity.

The protein expression of CYP2C19 showed no significant difference across all experimental groups, indicating that this enzyme may have a limited role in the development of alcohol-induced liver damage, or its influence on alcohol could be balanced by other regulatory mechanisms. These findings reveal the specific effects of chronic ethanol intake on the liver CYP450 subtypes.

### 2.4 Changes in AFLD rat liver microsome P450 enzyme activity

**FIGURE 5:**
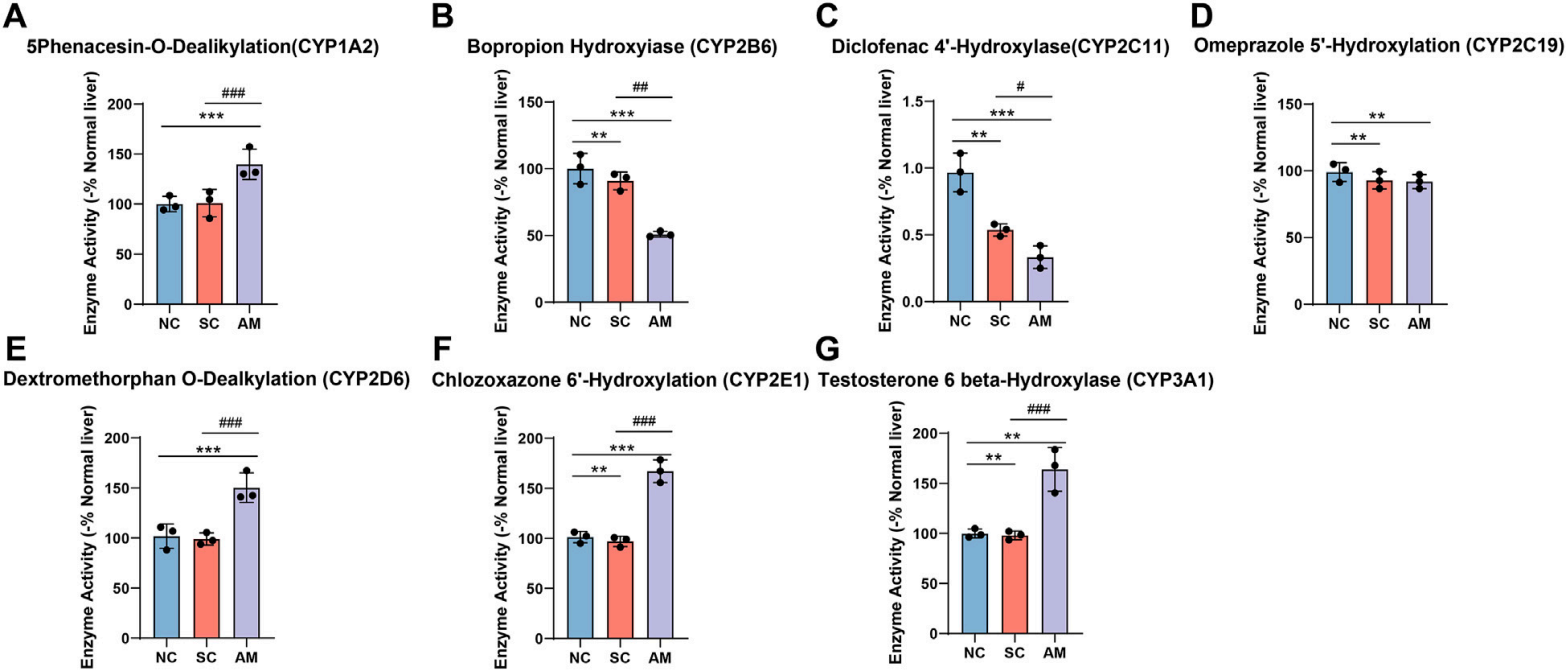
Enzyme activity of CYP450 enzymes in microsomes of AFLD rats. Note: **(A)** Enzyme activity of CYP1A2 in rat liver tissue; **(B)** Enzyme activity of CYP2B6 in rat liver tissue; **(C)** Enzyme activity of CYP2C11 in rat liver tissue; **(D)** Enzyme activity of CYP2C19 in rat liver tissue; **(E)** Enzyme activity of CYP2E1 in rat liver tissue; **(F)** Enzyme activity of CYP3A1 in rat liver tissue; **(G)** Enzyme activity of CYP3A1 in rat liver tissue. Compared to the normal control group, * indicates P < 0.05, ** indicates P < 0.01, and *** indicates P < 0.001, indicating significant differences as determined by the LSD test. Compared to the sucrose solution group, # indicates P < 0.05, ## indicates P < 0.01, and ### indicates P < 0.001, also determined by the LSD test. All experiments were repeated three times.

As shown in Figure 5, in line with the changes in mRNA and protein expression, the enzyme activity of CYP1A2, CYP2D6, CYP2E1, and CYP3A1 significantly increased in the AM group compared to the NC and SC groups. This increase reflects the liver’s enhanced metabolic capacity to cope with the demands of alcohol and its metabolites after long-term ethanol exposure. It also highlights the specific impact of ethanol intake on the liver’s CYP450 enzyme system, with notable decreases in CYP2B6 and CYP2C11, indicating a negative effect on these enzymes’ ability to metabolize other endogenous and exogenous compounds. Through methods such as microsome P450 enzyme activity assays, we conducted a detailed analysis of the activity of these enzymes. These data further confirm the complex regulatory effects of ethanol intake on the activity of different CYP450 enzyme subtypes in the liver.

### 2.5 Expression of ALDH2 and P-glycoprotein (P-gp) proteins in AFLD rats

**FIGURE 6:**
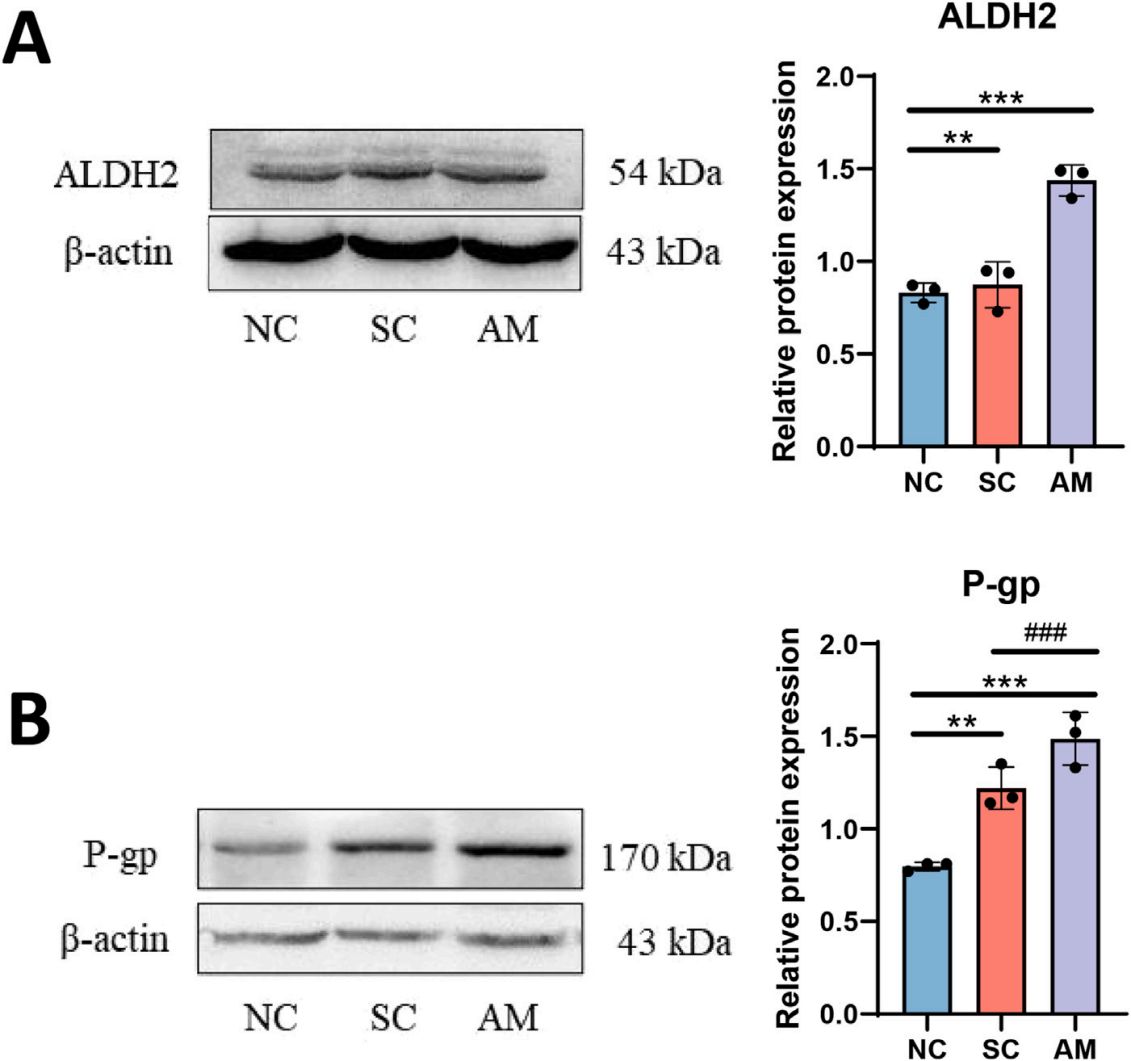
Effects of AFLD on the expression of ALDH2 and P-gp proteins in rats. Note: **(A)** Protein level of ALDH2 in rat liver tissue; **(B)** Protein level of P-gp in rat liver tissue. Compared to the normal control group, * indicates P < 0.05, ** indicates P < 0.01, and *** indicates P < 0.001, indicating significant differences as determined by the LSD test. Compared to the sucrose solution group, # indicates P < 0.05, ## indicates P < 0.01, and ### indicates P < 0.001, also determined by the LSD test. All experiments were repeated three times.

Compared with the NC and SC groups, the ALDH2 protein level was increased in the AM group (Figure 6A). ALDH2 is a key enzyme responsible for further oxidizing acetaldehyde in the ethanol metabolism process to convert it into harmless acetic acid, thereby reducing the detrimental effects of alcohol intake. In the context of AFLD, the activity and expression levels of ALDH2 are crucial in regulating alcohol-induced oxidative stress and mitigating liver damage. In the AM group with long-term ethanol intake, the increased ALDH2 protein level may reflect an attempt by the liver to upregulate ALDH2 expression to cope with acetaldehyde generated during ethanol metabolism in the progression of alcoholic fatty liver disease. However, this regulation may not be sufficient to fully counteract the harmful effects of alcohol. Additionally, we observed a significant increase in P-gp protein expression in the AM group (Figure 6B). This notable increase in P-gp protein expression reveals a different regulatory mechanism. As a transmembrane transport protein, P-gp protein plays a crucial role in drug excretion and metabolism, especially in maintaining the balance of drugs and other small molecules inside and outside cells. The upregulation of P-gp expression may be an adaptive response of the liver to long-term alcohol exposure, aiming to enhance the excretion of toxic substances and potential accumulated metabolites to protect liver cells from harm. This increase may reflect the liver’s effort to maintain its detoxification function and alleviate alcohol-induced toxicity. Therefore, the increased expression of P-gp is significant in the context of AFLD, not only because it reflects adaptive changes in response to alcohol toxicity in the liver but also because it affects the liver’s ability to process various drugs, which may impact the efficacy and safety of drug therapy in individuals with AFLD.

### 2.6 Variations in blood drug concentrations at different time points, pharmacokinetic analysis, and differences in metabolites

**FIGURE 7:**
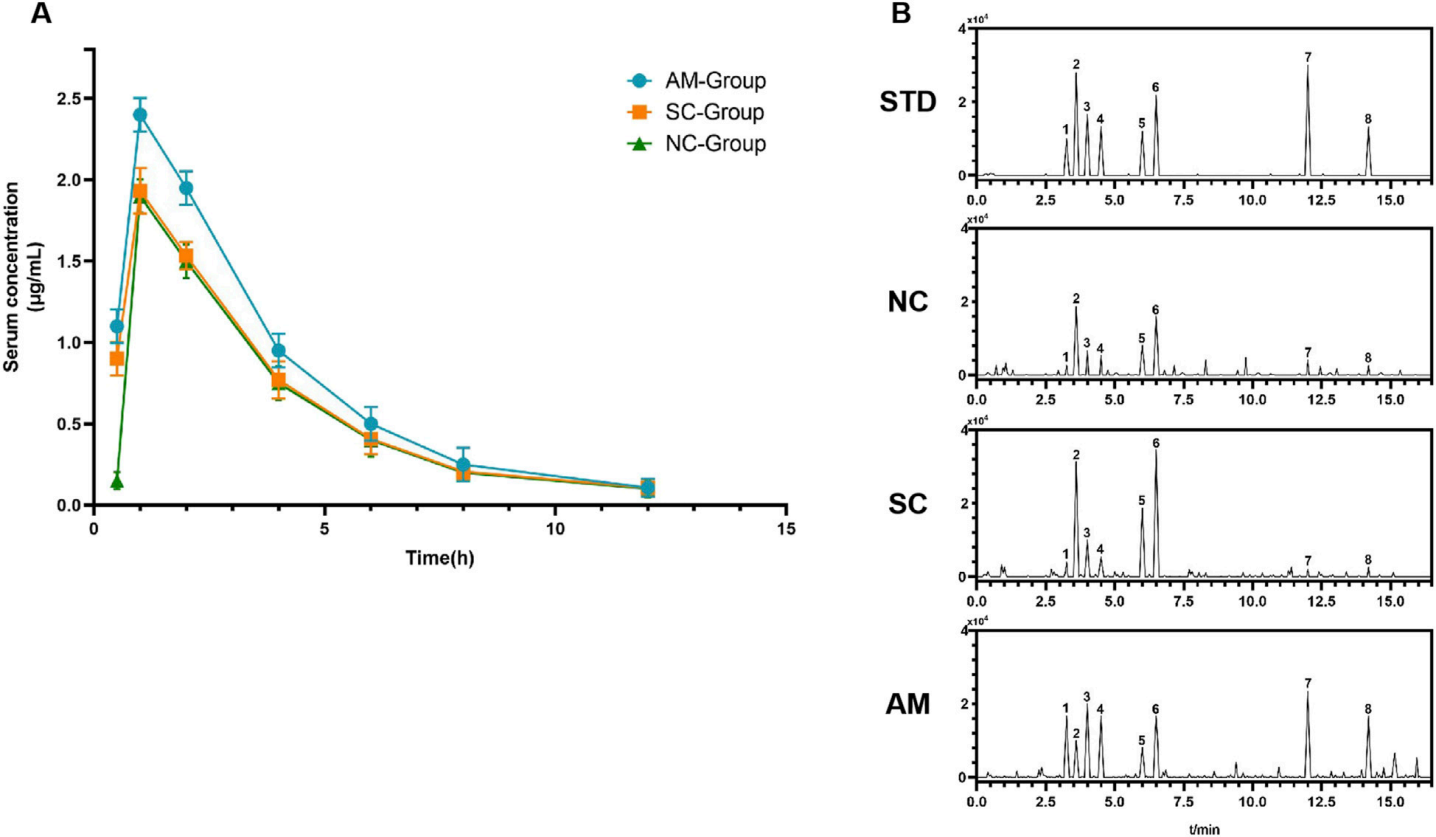
Serum drug concentration and metabolite profiles in different treatment groups after administration. Note: **(A)** Changes in serum drug concentration over time in the NC group (normal control), SC group (sucrose group), and AM group (ethanol group) post-administration. Data for each group are presented as mean ± standard error (n = 15). **(B)** High-performance liquid chromatography (HPLC) mass spectra of the rats in each group: STD: Standard spectrum used for peak identification of metabolites. NC: Mass spectrum of the control group showing baseline metabolite concentrations. SC: Mass spectrum of the sucrose group showing significant peaks for glucose and fructose as direct metabolic products of sucrose. AM: Mass spectrum of the ethanol group displaying high and sustained concentrations of ethanol and its main metabolites, acetaldehyde and acetic acid, as well as peaks for ethyl palmitate and ethyl oleate. Peaks for the main metabolites are labeled in the HPLC chromatograms as follows: 1-acetaldehyde, 2-pyruvic acid, 3-lactic acid, 4-acetic acid, 5-fructose, 6-glucose, 7-ethyl palmitate, 8-ethyl oleate.

Figure 7A shows the changes in serum drug concentrations at different time points after administration in the NC, SC, and AM groups. Statistical analysis revealed that the AM group exhibited a faster increase in drug concentration, with a significantly higher Cmax compared to the NC and SC groups, suggesting that long-term alcohol exposure may affect drug absorption or primary metabolism. Additionally, Supplementary Table S2 summarizes the main pharmacokinetic parameters, including Cmax, Tmax, AUC, and MRT. The MRT in the AM group was significantly higher (24.48 h, p < 0.01), indicating that alcohol may prolong the drug’s residence time by affecting hepatic metabolism or clearance. Furthermore, the AUC₀-∞ and AUC₀-L∞ were significantly increased in the AM group, suggesting higher systemic exposure to the drug. Tmax showed no significant difference among the groups (p > 0.05), indicating a limited effect of alcohol on drug absorption rate. In the pharmacokinetic study (Figure 7B), the mass spectrometry graph of the NC group shows baseline levels of metabolite concentrations without peaks of ethanol or its metabolites, indicating normal physiological metabolism in rats under no ethanol exposure conditions. The mass spectrometry graph of the SC group exhibits significant peaks of glucose and fructose as direct metabolites of sucrose, demonstrating a rapid metabolic response following sucrose intake and normal metabolic function. In contrast, the graph of the AM group is more complex, featuring high and sustained peaks of ethanol and its primary metabolites, acetaldehyde and acetic acid, as well as peaks of ethyl acetate and ethyl phosphate, indicating potential alterations in metabolic pathways and reduced metabolic rates due to prolonged ethanol exposure. These results highlight the significant impacts of different treatments on the metabolic status of rats.

### 2.7 Overexpression of CYP2C11 and CYP2B6 alleviates ethanol-induced AFLD

**FIGURE 8:**
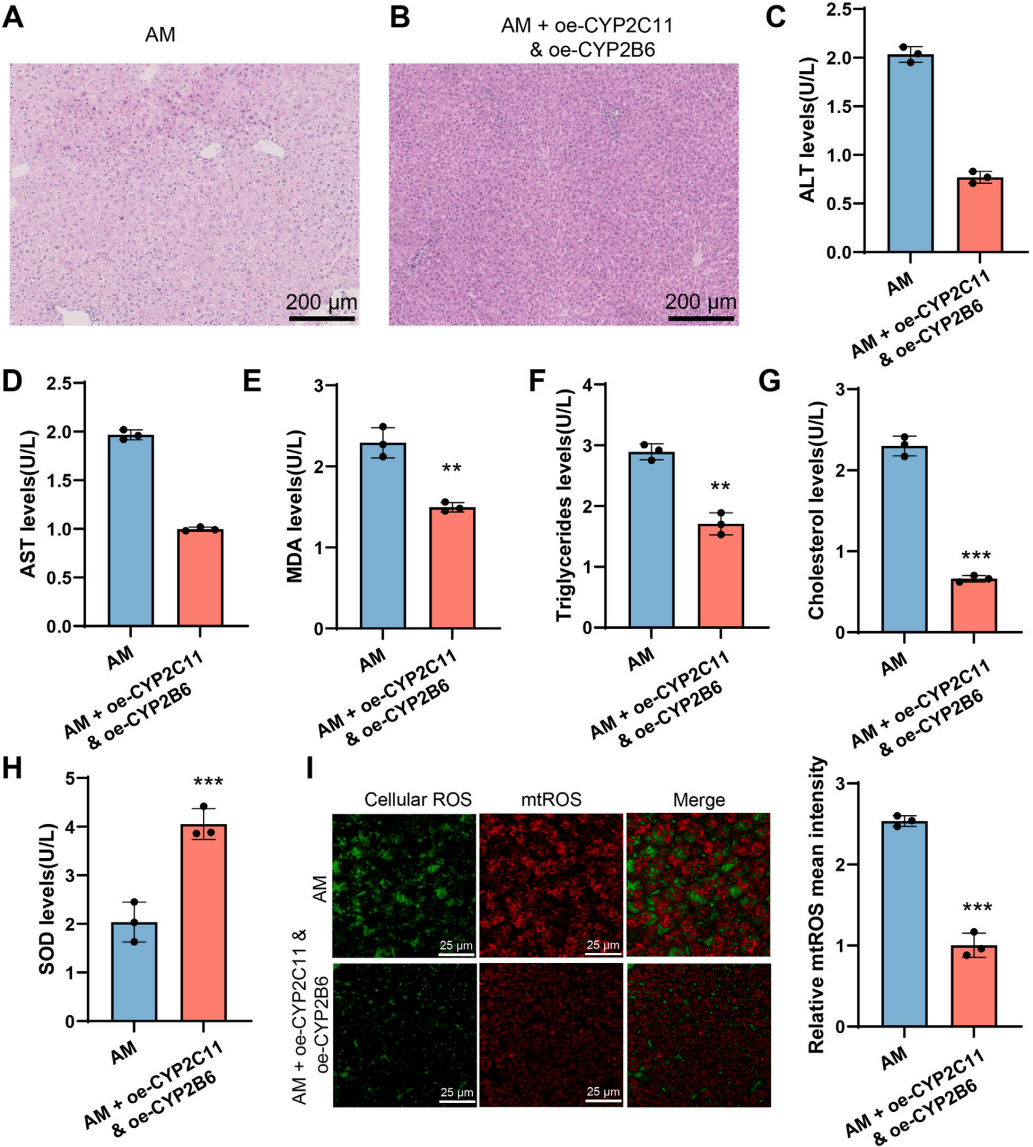
CYP2C11 and CYP2B6 Overexpression Reduces Lipid Accumulation and Oxidative Stress in AFLD. Note: **(A-B)** Severe tissue vacuolation and significant lipid droplet accumulation were observed in the ethanol intake group, while no lipid droplets were present in the group with simultaneous overexpression of CYP2C11 and CYP2B6. **(C–H)** Serum biochemical analysis. **(I)** ROS detection. All experiments were repeated three times. ***P < 0.001, **P < 0.01, *P < 0.05, indicating significant differences between groups.

To further confirm the effects of ethanol intake on hepatic function and drug metabolism mediated by different CYP450 enzyme subtypes, we performed a rescue experiment by overexpressing CYP2C11 and CYP2B6 simultaneously in AFLD model rats. The experimental results showed that overexpression of CYP2C11 and CYP2B6 alleviated hepatic lipid accumulation and reversed steatosis in the AM group (Figures 8A, B). Serum biochemical analysis revealed significant improvements in related indicators such as MDA and SOD (Figures 8C–H). Furthermore, ROS detection indicated that intracellular oxidative stress in the AM group was alleviated, with significantly reduced ROS levels following the overexpression of CYP2C11 and CYP2B6 (Figure 8I). These findings suggest that alcohol affects hepatic function and drug metabolism by modulating CYP450 enzymes.

## 3 Discussion

Long-term ethanol consumption is closely associated with the development of AFLD, as described in numerous previous studies (Niedzwiedz-Massey et al., 2022; Papaefthymiou et al., 2023; Freitas et al., 2022). In this study, through comparative analysis with existing research, we not only confirmed that ethanol intake significantly increases specific CYP450 enzyme subtypes’ expression and activity, such as CYP1A2, CYP2D6, CYP2E1, and CYP3A1, aligning with prior findings of ethanol-induced elevation in CYP2E1 enzyme activity, but also revealed for the first time a significant decrease in the activities of CYP2B6 and CYP2C11, addressing a gap in current literature in this field (Otani et al., 2005; Cicali et al., 2020). The increased activity of CYP2E1 reflects its pivotal role in ethanol metabolism, whereas the reduced activities of CYP2B6 and CYP2C11 may indicate the negative impact of ethanol intake on other hepatic metabolic pathways, potentially affecting the liver’s ability to process drugs and toxins. Moreover, distinct from previous studies, this research employed a comprehensive methodological approach, including mRNA expression analysis, quantification of protein levels, and direct measurement of enzyme activities, to provide a more comprehensive and detailed description of the changes in the CYP450 enzyme system. Possible sources of these differences may stem from variations in experimental methods, the animal models utilized, or levels of ethanol exposure, all of which could influence the observations of CYP450 enzyme subtype expression and activity results.

**FIGURE 9:**
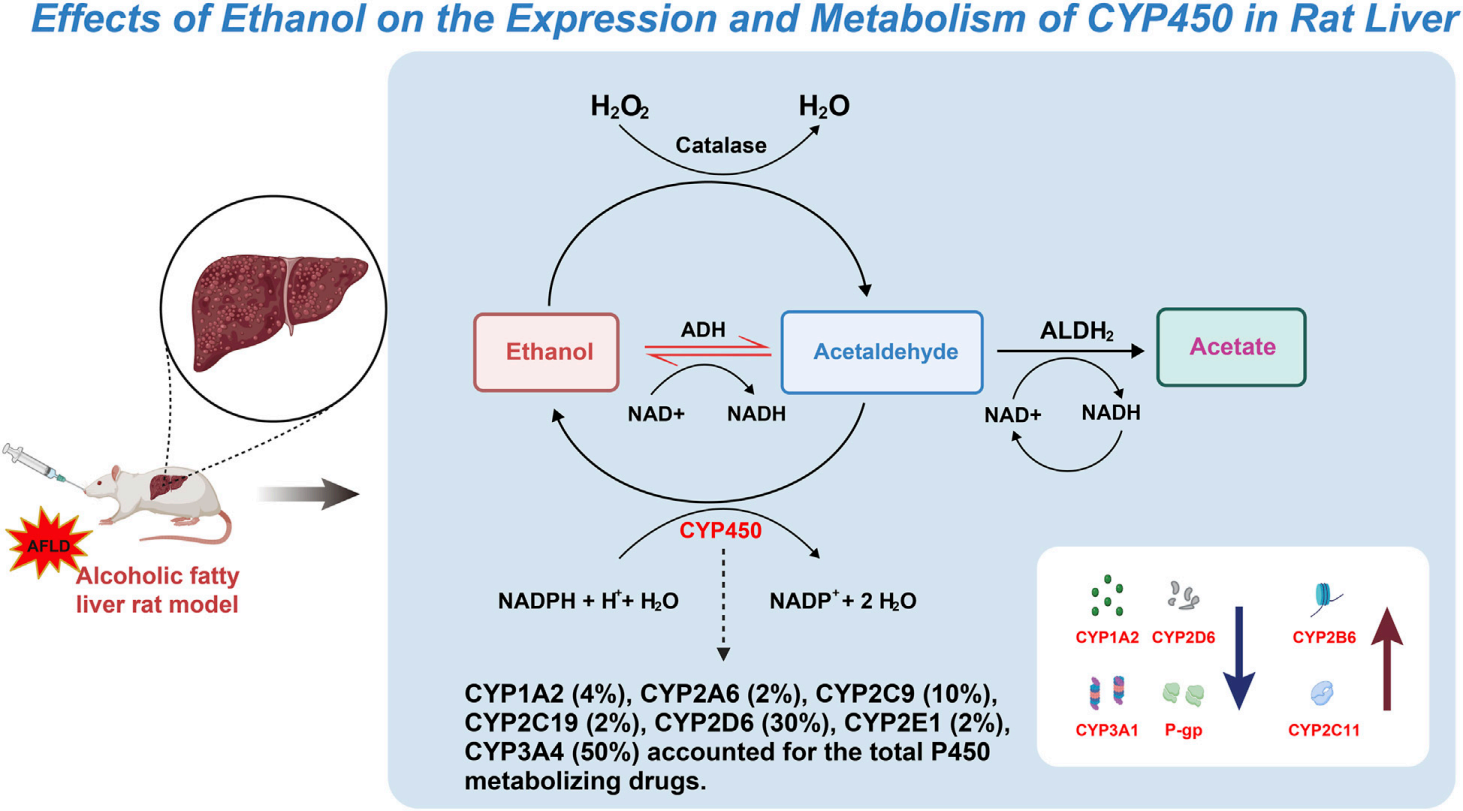
Mechanistic pathway map of the effect of ethanol on CYP450 expression and metabolism in rat liver.

The expression and activity variations of specific CYP450 subtypes play a crucial role in understanding the molecular mechanisms of AFLD (Jiang et al., 2024; Rabelo et al., 2024). The impact of long-term ethanol consumption on these enzyme subtypes reveals their intricate role in hepatic metabolism, subsequently affecting AFLD development (Guo et al., 2022; Fromenty and Roden, 2023; Rinella et al., 2023). The findings of this study, particularly the increased activities of enzyme subtypes such as CYP1A2 and CYP2D6, along with the decreased activities of CYP2B6 and CYP2C11, offer new insights into how ethanol alters liver function through modulation of the CYP450 enzyme system. Additionally, the increased expression of the P-gp protein further indicates that ethanol may also intervene in normal liver function through other mechanisms, such as affecting transport proteins involved in drug metabolism and excretion (Figure 9).

The molecular mechanisms behind the increase or decrease of specific CYP450 enzyme subtypes in AFLD models are primarily related to ethanol metabolism (Qiang et al., 2021). Ethanol is metabolized to acetaldehyde by alcohol dehydrogenase (ADH), which is further oxidized to acetic acid by ALDH2. However, ethanol can also undergo metabolism through the CYP450 enzyme system, particularly CYP2E1 (Xu et al., 2022; Wang et al., 2021a). The increased activity of CYP2E1 not only accelerates ethanol metabolism but also enhances the generation of ROS, leading to oxidative stress and cellular damage, a critical factor in AFLD development. The increased activities of CYP1A2 and CYP2D6 may represent adaptive responses to ethanol-induced oxidative stress to bolster hepatic detoxification capacity. Nevertheless, this heightened metabolic activity may somewhat exacerbate oxidative stress, creating a vicious cycle (Asejeje et al., 2023).

On the contrary, the decreased activities of CYP2B6 and CYP2C11 may reflect compromised hepatic metabolic capacity under prolonged ethanol exposure (Pang et al., 2024). As these enzyme subtypes are involved in the metabolism of numerous drugs and endogenous compounds, their reduced activities may lead to accumulating these substances in the body, increasing the risk of toxic reactions. Additionally, such changes in CYP450 enzyme activity could also impact lipid metabolism, thereby promoting fat accumulation in the liver and exacerbating the pathological severity of AFLD (Nasrin et al., 2021; Mangó et al., 2022; Li et al., 2023a).

The increase in P-gp protein expression reveals another liver functional regulatory mechanism induced by ethanol intake (Silva et al., 2021). As a crucial transmembrane transport protein, P-gp participates in the excretion of multiple drugs (Mora Lagares et al., 2019). Its increased expression may represent a mechanism employed by the liver to mitigate the toxicity of ethanol and its metabolites by accelerating the excretion of these substances to protect cells from damage (Imazio and Nidorf, 2021; Iversen et al., 2022; Balasubramanian and Maideen, 2021). However, this may also impact the pharmacokinetic properties of other drugs, leading to treatment failures or drug toxicities (Gomez-Sanchez and Gomez-Sanchez, 2023; Chen et al., 2023; Hohmann et al., 2023). The alterations in specific CYP450 enzyme subtype expression and activity and the increase in P-gp protein expression reflect the liver’s multifaceted adaptive regulation and coping strategies under prolonged ethanol exposure (Nagai et al., 2023). These regulatory mechanisms aim to shield the liver from the toxic damage of ethanol and its metabolites, yet they may also exacerbate AFLD development due to changes in metabolic pathways (Ishteyaque et al., 2023; Hussain et al., 2022).

The increased ROS production resulting from ethanol-induced elevation in CYP2E1 activity not only directly damages liver cells but may also modulate inflammatory responses and cell apoptosis by affecting other cellular signaling pathways, such as NF-κB and MAPK pathways (Osna et al., 2022; Yan et al., 2023b; Luo et al., 2021). Furthermore, alterations in the CYP450 enzyme system may affect hepatic lipid metabolism, as excessive activity of CYP2E1 can promote lipid peroxidation, while decreased activity of CYP2B6 and CYP2C11 may slow normal lipid metabolism, collectively promoting fat accumulation in the liver. Moreover, while the increased expression of P-gp protein may initially help reduce ethanol toxicity, long-term effects could impair the liver’s ability to process other drugs and toxins, increasing the risk of drug interactions and potentially affecting hepatic metabolism by altering the transport and metabolism of bile acids (Stöllberger and Finsterer, 2023; Zhang et al., 2023b).

The changes in the expression of these CYP450 enzyme subtypes and P-gp protein reflect the impact on hepatic metabolism and AFLD progression, showcasing the liver’s complex adaptive response to long-term ethanol exposure (Zheng et al., 2023; Gomez-Sanchez and Gomez-Sanchez, 2023; Zhai et al., 2023). It offers new insights into the pathogenesis of AFLD and underscores the potential value in developing therapeutic strategies targeting specific CYP450 enzyme subtypes and P-gp proteins (Zeng et al., 2019; Suzuki et al., 2020). For instance, in cases of elevated CYP2E1 activity, specific inhibitors or antioxidants could be developed to reduce ROS production and lipid peroxidation; for lipid metabolism disturbances caused by reduced CYP2B6 and CYP2C11 activities, drug modulation or supplementation with specific nutrients may restore normal function (Ishteyaque et al., 2023; Ye et al., 2021). The AM group exhibited higher Cmax and lower clearance after long-term alcohol exposure, possibly due to alcohol-induced changes in liver metabolism, reducing primary drug metabolism (Long et al., 2018; Zhang et al., 2021). It was supported by statistical analysis of pharmacokinetic parameters, where the AM group also showed differences in drug bioavailability (Barreto et al., 2023; Shekar et al., 2023; Gera et al., 2022). These findings align with the mass spectrometry analysis in Figure 7B, where the AM group demonstrated elevated and sustained peaks of ethanol and its main metabolites, acetaldehyde and acetic acid, along with secondary metabolites like fatty acid ethyl esters and phosphoric acid ethyl esters, indicating altered metabolic pathways and reduced metabolic rates (Lee et al., 2023; Hu et al., 2023).

While this study offers important insights into the impact of ethanol intake on the expression and activity of CYP450 enzyme subtypes, there are still some limitations. Specifically, our understanding of the specific mechanisms by which ethanol influences the activity of CYP450 enzyme subtypes is not sufficiently deep, particularly at the level of cellular signal transduction and transcriptional regulation. Additionally, although we used animal models to simulate human AFLD, physiological and metabolic differences may exist between animal models and human diseases, potentially limiting the direct extrapolation of study results to humans. Furthermore, this study primarily focused on liver research without considering how alcohol may affect the activity of CYP450 enzymes in other organs, which could have implications for overall metabolism.

Future research could benefit from introducing more diverse animal models and applying advanced molecular biology techniques, such as CRISPR gene editing and single-cell sequencing, to precisely elucidate the effects of alcohol intake on liver cells. Simultaneously, conducting more clinical studies will be crucial for validating our research findings and advancing the development of new therapeutic strategies, especially in personalized and precision medicine, where investigating differences in individual responses to alcohol may lead to new therapeutic targets. In conclusion, this study lays a solid foundation for further exploring the molecular mechanisms of AFLD and may pave the way for establishing theoretical groundwork for developing more personalized and effective treatment strategies.

## 4 Materials and methods

### 4.1 Ethical statement

This study strictly adhered to the ethical guidelines for animal experiments and relevant regulations. Our Institutional Animal Ethics Committee approved all animal experimental procedures under the ethical approval number “LLSC20230906” The selection, breeding, farming, and disposal of animals complied with internationally recognized guidelines for using and caring for laboratory animals. Experimental rats were housed in a controlled environment with constant temperature, humidity, and a 12-h light/dark cycle, provided ample food and water. All experimental manipulations were performed under the supervision of skilled professionals proficient in laboratory animal welfare to minimize animal distress and discomfort to the greatest extent possible. All procedures for the termination of experiments were carried out under strict anesthesia to ensure that rats did not experience any pain.

### 4.2 Bioinformatics analysis

Transcriptomic data related to “AFLD” was retrieved from the GEO database (https://www.ncbi.nlm.nih.gov/geo/) using the keyword search. The species selected for analysis was rats, and the chip GSE66316, with the highest number of samples, was chosen as the research subject. This chip consists of 6 healthy control rats and 6 rats with fatty liver. Clustering analysis of gene expression patterns was performed using the “pheatmap” package in R language, employing Euclidean distance and complete linkage clustering methods to demonstrate gene similarities and analyze the correlation between differentially expressed genes (DEGs) and the disease. Analysis used the “DisGeNET” database and its corresponding R package, focusing on AFLD as the query condition using default parameters. Differential analysis of gene expression profiles in the two group rats was performed with the GEO2R tool, with the threshold set at |logFC| > 0.7 and p-value <0.05 for DEGs selection. The results of the differential analysis were visualized using the “ggplot2” package in R. Gene Ontology (GO) and Kyoto Encyclopedia of Genes and Genomes (KEGG) enrichment analysis of DEGs was performed using the “clusterprofiler” package in R, with a threshold set at p < 0.05 for selecting enriched terms. Enriched terms were searched, and relevant genes and network diagrams were obtained from the STRING website, with a minimum interaction score threshold set at 0.4 to ensure that interactions within the network are highly confident. Cytoscape software was utilized for further network analysis and visualization, and the “MCODE” plugin was used to identify highly interconnected modules within the network.

### 4.3 Establishment of an AFLD rat model

Sixty healthy male Sprague-Dawley rats (SD-2024-RAT-045, Beijing Vital River Laboratory Animal Technology Co., Ltd., Beijing, China) weighing 200–250 g were selected. The animals were housed under a 12-h light/dark cycle at a constant laboratory temperature of 22°C ± 2°C and relative humidity of 50% ± 10%, with access to ample food and water. Sixty rats were randomly assigned to four groups (fifteen rats per group): the normal control group (NC group), the sucrose solution group (SC group), the ethanol intake group (AM group), and the ethanol intake group with simultaneous overexpression of CYP2C11 and CYP2B6 (AM + oe-CYP2C11&CYP2B6 group). The NC group received an equal volume of normal saline daily via oral gavage. The SC group was given an 18% sucrose solution (w/v) and an equal volume of normal saline instead of ethanol. The AM group started with a 5% ethanol concentration, increasing by 5% weekly to 40% while maintaining a constant 18% sucrose concentration (v/v). The plasmid vector for gene overexpression, pCMV6-AC-GFP (LM-2069, LMAI Bio, Shanghai, China), was used to construct CYP2C11 and CYP2B6 plasmids, which were developed by Sangon Biotech (Shanghai, China). After constructing the AM rat model, overexpression plasmids were transfected into lentiviruses via tail vein injection (Liu et al., 2020). The lentivirus injections (5 × 10^7^ IU/rat) were administered twice, with a minimum interval of 2 days between injections, starting on day 7 after the initiation of sucrose treatment (Ma et al., 2020).

The daily ethanol and sucrose feeding volumes were weight-adjusted for the rats, approximately 20 mL per kilogram of body weight, and continued for 12 weeks. Weekly monitoring of rat weight, observation and recording of appetite, general health status including activity level, fur condition, and behavioral changes were documented. Daily feeding amounts were meticulously recorded to accurately adjust ethanol and sucrose solution volumes. After 12 weeks, the rats were euthanized by cervical dislocation following unconsciousness induced by carbon dioxide (CO₂) inhalation. Liver tissues were dissected and stored at −80°C for subsequent histological and molecular biology analyses. Blood samples were collected, and serum was separated by centrifugation (3,000 rpm, 4°C, 15 min) and stored at −80°C for future use.

### 4.4 Serum biochemical analysis

Serum-related indicators were measured using commercial assay kits (BioSino Biotechnology and Science Inc., Beijing, China) following the manufacturer’s protocols. Specifically, serum samples, substrates, and buffer solutions were prepared according to the kit instructions. The reaction mixture was incubated at 37°C for 20 min, and absorbance was measured at 340 nm to record changes. Concentrations were calculated based on a standard curve (Li et al., 2018).

### 4.5 Detection of total ROS and mitochondrial ROS

Reactive oxygen species (ROS) levels were detected using the fluorescent dye 2′,7′-dichlorodihydrofluorescein diacetate (DCF) (HY-D0940, Abcam, United Kingdom). A DCFDA (10 mM) stock solution was prepared in methanol and diluted in a culture medium to a working concentration of 100 μM. Endothelial cells (2 × 10^4^ cells) were seeded on coverslips in six-well plates and cultured overnight. The cells were treated with H_2_O_2_ (200 μM) the next day for 24 h. After treatment, the coverslips were washed with ice-cold Hank’s Balanced Salt Solution (HBSS) (H8264, Sigma Aldrich, Shanghai, China) and incubated with 100 μM DCFDA at 37°C for 30 min. After washing, the coverslips were mounted onto slides and imaged using a multiphoton confocal microscope (A1R, Nikon, United States) with a ×100 objective. Images were analyzed using Nikon Imaging Software NIS-Element.

Mitochondrial superoxide compounds (SOX) were assessed using mito-SOX Red (M36008, Thermo Fisher, United States). Endothelial cells (2 × 10^4^ cells) were seeded on coverslips placed in six-well plates and cultured overnight. The cells were treated with H_2_O_2_ (200 μM) the next day for 24 h. After treatment, the coverslips were washed with ice-cold 1 × PBS and incubated at 37°C with 2 μM mito-SOX working solution for 30 min. Following incubation, the coverslips were washed with 1 × PBS, mounted onto slides, and imaged using a multiphoton confocal microscope with a ×100 objective. Images were analyzed using Nikon Imaging Software NIS-Element (Maity et al., 2022).

### 4.6 Pharmacokinetic experiment

Rats were administered orally with the same doses of normal saline and ethanol in the NC, SC, and AM groups, following the same method. After 12 weeks, blood samples were collected from the rats at specified time points (0.5, 1, 2, 4, 6, 8, and 12 h post-dosing) during the final dosing. The blood samples were centrifuged to separate serum and then stored at −80°C for further use.

Utilizing liquid chromatography-mass spectrometry (LC-MS) technology, the concentrations of ethanol and its metabolites in serum samples were analyzed. Analyze the pharmacokinetic parameters of the drug, such as the maximum plasma concentration (Cmax), the mean residence time (MRT) of ethanol in the body, and the bioavailability of ethanol. Data processing was conducted using specialized software XCMS, involving peak identification, integration, and normalization. Multivariate statistical analyses were performed on metabolite data to reveal metabolic differences between groups, such as principal component analysis (PCA) and orthogonal partial least squares discriminant analysis (OPLS-DA). Metabolites were annotated and compared with known databases (HMDB, METLIN) to confirm their identities. Differences in pharmacokinetic parameters among the NC, SC, and AM groups were compared to evaluate the effects of alcohol exposure and CYP450 enzymes, aiming to identify metabolite variances.

### 4.7 Hematoxylin and eosin (H&E) staining

At the end of the experiment, the animals were euthanized to obtain liver samples. The liver was excised and immediately fixed in a 4% paraformaldehyde solution. Subsequently, the liver tissues underwent dehydration using a series of progressively concentrated alcohol solutions, with each step lasting approximately 1–2 h. Following fixation, tissue samples were cleared (commonly with xylene) and embedded in paraffin for sectioning. The embedded liver tissues were cut into continuous slices of 4–5 μm in thickness using a microtome (RM2235, Leica, Germany) and transferred onto glass slides for drying in a 60°C oven. Dewaxing and hydration procedures were then performed, involving the removal of paraffin with xylene and hydration with a series of decreasing concentrations of alcohol solutions. The dewaxed sections were subsequently stained with hematoxylin and eosin to visualize cell nuclei and cytoplasmic structures, followed by dehydration, clearing with xylene, and mounting. For analysis, the sections were observed under an optical microscope (such as those manufactured by Leica, Germany).

### 4.8 RT-qPCR

Total RNA was extracted from rat liver tissue using TRIzol reagent (15596026, Invitrogen, United States). cDNA was synthesized using the cDNA synthesis kit (RR036A, Takara, Japan). Specific gene expression was detected by combining the PCR master mix (206143, Qiagen, United States) with the Pikoreal 96 real-time PCR system (QPCR-96X, Thermo Scientific, United States). Quantitative PCR analysis was performed for CYP450 enzyme-related genes with the endogenous gene β-actin as a reference, and the relative mRNA expression levels were quantified by calculating the 2^−ΔΔCt^ values. All experiments were repeated three times.

### 4.9 Western blot

Liver samples were lysed using RIPA lysis buffer (P0013B, Beyotime, China). The total protein concentration was evaluated using the BCA protein assay kit (AR0146, Doctor’s Scientific Inc., China). SDS-PAGE was employed to separate total proteins, followed by protein transfer onto pre-wetted PVDF membranes with methanol (IPVH00010, Millipore Corp, United States). Non-specific protein blocking was carried out at room temperature for 3 h using TBST solution (BL315B, Boist, China) containing 5% milk. The target proteins were probed with primary antibodies overnight on nitrocellulose membranes, followed by three washes with TBST/Tween 20 (0.075%) for 15 min each and subsequent incubation with labeled secondary antibodies for 1 h before washing. Protein visualization was performed using the Millipore Immobilon Western Chemiluminescent HRP substrate. The primary antibodies used in the experiments were polyclonal rabbit anti-human CYP450CYP1A2, 2B6, 2C11, 2C19, 2E1, 2D6, 3A1 (bs-20720R, Bioss, China) at a 1:300 dilution. The secondary antibodies, goat anti-mouse or anti-rabbit horseradish peroxidase conjugates, were diluted at 1:10,000 in TBST containing 5% milk. β-actin served as the loading control. The experiments were repeated three times.

### 4.10 Isolation of rat liver microsomes

Rat liver microsomes were isolated using differential centrifugation. Each sample was mixed with 50 mM Tris-HCl buffer, homogenized, and then centrifuged at 10,000 × g for 30 min. The supernatant was centrifuged at 100,000 × g for 1 h using a high-speed centrifuge (Himac CP100WX, Hitachi, Japan). The quality of microsomes was confirmed by assessing protein concentration and enzyme activity.

### 4.11 Microsomal P450 enzyme activity measurement

The activities of CYP1A2, CYP2B6, CYP2C19, CYP2C11, CYP2E1, CYP2D6, and CYP3A1 were quantitatively determined using specific labeled substrates and an NADPH generation system containing 100 mM potassium phosphate buffer, 1 mM NADP (N1630, Sigma, United States), 10 mmol/L G-6-P (G7877, Sigma, United States), 0.1 U/mL G-6-PDH (G8529, Sigma, United States), and 5 mM MgCl2. The NADPH generation system was added to microsomes (total protein concentration of 1 mg/mL) with a total volume of 190 μL. Each reaction system was supplemented with 10 μL of substrate. Enzymatic reactions were terminated by adding 600 μL of acetonitrile. Product concentrations were determined by validated liquid chromatography/tandem mass spectrometry.

To ensure the statistical significance of the research findings and avoid overinterpretation, sample size and statistical power analyses were conducted using GPower software. Experiments were repeated at least three times, and the sample size was calculated based on the expected effect size, α-error (typically set at 0.05), and β-error (usually set at 0.2, corresponding to 80% statistical power).

### 4.12 Statistical analysis

A series of statistical analyses were performed using the SPSS 13.0 software package to comprehensively analyze the impact of chronic ethanol consumption on rat liver CYP450 activity. Descriptive statistical analysis was initially conducted, including calculating the mean, standard deviation, median, and quartiles. The normality of the data was assessed using the Shapiro-Wilk and Kolmogorov-Smirnov tests. A one-way analysis of variance (ANOVA) was applied to data that met the assumptions of normal distribution and homogeneity of variances, followed by Tukey’s Honestly Significant Difference (HSD) test for post hoc multiple comparisons. Non-normally distributed data were analyzed using the Kruskal-Wallis test. The homogeneity of variances was evaluated using Levene’s test. Depending on the data distribution, Pearson correlation (parametric) or Spearman correlation (non-parametric) analyses were selected for assessing correlations, while linear regression analysis was used to explore relationships between continuous variables. The significance level for all statistical tests was set at 0.05.

## Data Availability

The original contributions presented in the study are publicly available. The transcriptomic data can be found at GEO: https://www.ncbi.nlm.nih.gov/geo/query/acc.cgi?acc=GSE66316. The metabolomic data can be found at ProteomeXchange with the identifier PXD063375 (http://proteomecentral.proteomexchange.org/cgi/GetDataset?ID=PXD063375) and at iProX with the identifier IPX0011826000 (https://www.iprox.cn/page/project.html?id=IPX0011826000).
