# Supplementary material for: Chronic alcohol intake disrupts cytochrome P450 enzyme activity in alcoholic fatty liver disease: insights into metabolic alterations and therapeutic targets
Source: Front Chem. 2025 May 13;13:1509785. doi: 10.3389/fchem.2025.1509785 (PMC12106329; doi:10.3389/fchem.2025.1509785)
Supplement: Supplementary file 1 [file Table2.docx]

**Table S2. Comparison of pharmacokinetic parameters between NC: SC and AM groups in a rat model after ethanol exposure.**

| **Group** | **T1/2 (h)** | **Cmax (µg/mL)** | **Tmax (h)** | **AUC0-L∞ (µg*h/mL)** | **AUC0-∞ (µg*h/mL)** | **T1/2 el (h)** | **MRT (h)** |
| --- | --- | --- | --- | --- | --- | --- | --- |
| NC Group Mean | 2.4 | 1.9 | 4.75 | 21.4 | 22.4 | 14.2 | 20.4 |
| SC Group Mean | 2.35 | 1.89 | 4.68 | 22.3 | 21.3 | 13.5 | 19.8 |
| AM Group Mean | 2.88 | 2.375 | 4.76 | 27.82 | 29.12 | 17.04 | 24.48** |

**Note:** *p < 0.05, **p < 0.01 compared to NC group. NC Group: Normal Control Group. SC Group: Sucrose Solution Group. AM Group：Alcohol Intake Group. T1/2: half-life. Cmax: maximum plasma concentration. Tmax: time to reach maximum plasma concentration. AUC0-L∞: area under the plasma concentration-time curve from time zero to the last measurable concentration. AUC0-∞: area under the plasma concentration-time curve from time zero to infinity. T1/2 el: elimination half-life. MRT: mean residence time.
